# Listening to Contemporary Art Music: A Morphodynamic Model of Cognition

**DOI:** 10.5334/joc.280

**Published:** 2023-07-04

**Authors:** Riccardo D. Wanke

**Affiliations:** 1 Centre for the Study of the Sociology and Aesthetics of Music –CESEM, Nova University Lisbon, Portugal

**Keywords:** Sound-based Music, Embodied cognition, Auditory Gestalt, Cross-modal Perception, Morphodynamic Theory

## Abstract

This paper proposes that the perceptual and cognitive mechanisms involved when listening to certain genres within “sound-based” music, such as post-spectralism, glitch-electronica, and electroacoustic music and in various areas of sound art, are best understood within a connectionist cognitive framework described by morphodynamic theory. By analysing the specific characteristics of sound-based music, it is explored how this kind of music works at perceptual and cognitive levels. The sound patterns found in these pieces engage listeners more readily at a phenomenological level rather than through establishing long-term conceptual associations. They consist of a set of geometries in motion appearing to the listener as “image schemata”, as they embody Gestalt and kinaesthetic principles portraying the forces and tensions of our being in the physical world (e.g., figure-background, near-far, superimposition, compulsion, blockage). In applying morphodynamic theory to the listening process involved in this kind of music, this paper discusses the results of a listening survey designed to investigate the functional isomorphism between sound patterns and image schemata. The results suggest that this music can be seen as a mean term within a connectionist model between the acoustic-physical world and the symbolic level. This original perspective opens up new pathways to access this kind of music and leads to a more general understanding of today’s modes of listening.

## Introduction

The perceptual aspect of sound has been the subject of an immense range of studies in the cognitive sciences, from psychology to neuroscience. In the field of music perception, the majority of these studies move between analytical surveys, which explore how we perceive sonic qualities and timbre of often simple sound types (*i.e*. single tones or noises) (Giordano & McAdams, 2010; Grey, 1977), and studies that have more to do with philosophy, semiology, and psychology (Kivy, 1991; Davies, 1994; Scruton, 1997; Ridley, 2004). Within this large group of studies on music perception, only a small number deal with the contemporary art music and experimental scenes (Deil et al., 2022; Mencke et al., 2019; Glover et al., 2018; Ockelford & Sergeant, 2012; Frey et al., 2009). This scarcity of research is paradoxical if we consider that, quite often, experimental music practices are themselves concerned with these very questions of perception.

Traditional approaches commonly used to study the perceptual mechanisms involved in music listening (Huron, 2006; Juslin & Sloboda, 2013; Snyder, 2001; Lerdahl & Jackendoff, 1983) are quickly found to be ineffective when applied to experimental music practices.^1^ Certain publications suggest original outlooks that aspire to expand the horizons of these conventional approaches (Kozak, 2020; Voegelin, 2014; Larson, 2012; Leman, 2008). What happens when music does not use tonal constructions, traditional narrative structures, or linear time perception, and contains instead noises, real-world sounds, a mix of acoustic and electronic sounds, or manipulated sounds, all potentially in combination and within non-linear structures, what is then our neurological and emotive response? Various genres within contemporary art music focus on specific characteristics of sound –including extended temporal forms and frequency ranges, aural stimuli at the threshold of our perception, and the use of unconventional listening environments– and due to these specific traits, the perception of this type of music may operate differently in so far as it confronts the listener with a wider range of sounds and evolutions compared to the more restricted set of conventional musical sounds (Imberty, 2005; Grisey, 1987).

This paper will focus on a particular set of music genres including electroacoustic music, mixed-source works, post-spectralism, glitch-electronica, and various areas of sound art. I choose the label “sound-based music” (Landy, 2007) –a term that is admittedly reductive but undeniably useful– to refer to currents in each genre possessing particular common features. Works within each of these genres show a range of characteristics such that, for example, not all electroacoustic music fits the “sound-based” label. At the same time, certain works across these genres share enough in common that a distinct cross-genre group can be identified. Works that fit this label do not focus on organized or dialectic constructions (i.e., melodies), rather they show an explicit interest in the intrinsic properties of sound and the creation of sonic textures and masses in motion, an interest that is attuned to how these are experienced. These pieces distinctly show a passage from sound seen as a *constituted* element of musical phrases with a given function to sound as a *constituent* element of music, autonomized in such a way that we may define it not through its usage but through its nature (Bonnet, 2012, p. 130). This cross-genre area of music has recently become more distinct due to its specific sonic features which call for new and comprehensive perspectives (Solomos, 2019; Bonnet, 2012; Wanke, 2021). This music is essentially perceived as a sequence of sound patterns which engage us more readily at a phenomenological and sensorial level rather than through establishing long-term conceptual associations. The musical material occupies, and even exceeds, the entire range of human hearing,^2^ exploring its dimensions (time, frequency, intensity) in a continuous and extended way through the use of unpitched tones, extreme dynamic contrasts, inharmonic spectra and noise. Lacking linear construction, narrative and temporality, such music challenges common expectations, exposing listeners to a series of stimuli which bring to mind a set of geometries –lines, planes, layers, 3D shapes– in motion. Recently, sound-based music has had an impact across an immense range of mainstream fields of music (movie and game soundtracks, sound design for advertising, and popular music from rap to dance electronica) often characterized by multisensory environments and virtual or augmented listening experiences. Hence, the challenge for the listener and the analyst concerns how we interact with this music that does not use standard musical forms but equally isn’t simply a catalogue of environmental recordings. It is worth taking a step back to look at our universal processing of all types of sound and to consider the broader theory of perception and cognition in relation to music. In doing this, we should discard the traditional belief that an aesthetic experience can only be associated with high-order cognitive processes and consider low-level perceptual steps as well (Santarcangelo & Wanke, 2020).

This article does not aim to compare the listening experiences between different music genres (Dean & Bayles, 2016, Mencke et al., 2022), but rather to explore how sound-based music works and its cognitive potential. By using analytical, theoretical and empirical approaches, I propose that morphodynamic theory (Thom, 1983; Petitot, 1985) can illuminate and efficiently describe the listening experience of sound-based music. First, I clarify the distinctiveness of sound-based music which makes it the perfect candidate to be studied within a morphodynamic framework. Morphodynamic theory, i.e. a connectionist model of cognition that bridges between the morphology of the external natural world and our internal cognitive structures, helps to understand how we can have sense of the sound patterns of this music as shapes in motion which represent tensions and forces. Then, in discussing the results of a listening survey, I explore how a morphodynamic model can be applied to an empirical study where I investigate the isomorphism between the sound patterns typical of this music and the evoked mental representations that I define as *image schemata*.

What emerges from the integration of the theoretical discussion and the empirical survey is the idea that sound-based music, due to its characteristics, embodies natural structures of motion (tensions, forces, shapes) that are at the core of our everyday experience of the world. Given this, the listening experience of this music can be efficiently understood within a connectionist model of cognition, bridging external and symbolic structures.

## Looking for new cognitive models for sound-based music

If we consider pieces by figures such as Annea Lockwood, Alvin Lucier, Jacob Ullman, Georg Friedrich Haas, Giovanni Verrando, Jürg Frey, Ryoji Ikeda, Jacob Kirkegaard, or Pan Sonic, we encounter a set of works whose sound material spills over the limits of our perception. These composers explore hypnotic, bewildering effects, constructing sculptural arrangements which engage listeners on a sensorial and phenomenological level. They have a unified non-dialectic understanding of sound, aiming to provide listeners with new perceptual paradigms. Retention and protention of the sonic flow in these compositions are intentionally put to the test (Glover et al., 2018). These compositions use sonic materials that exceed the definite form of a musical (or sound) object and that do not possess clear edges (being potentially confused with environmental sound or creating acoustic challenges in perception). By using primordial structures, these pieces thwart our predictive constructions and change our usual sense of expectation of future events. If we consider Frey’s string quartets (e.g., the second and third), we find a uniform sequence of suspended sounds. The aim of Frey in these pieces is to condense our processing of sonic streams to its essentials, favoring a static apprehension of sounds (cfr. “vertical listening”, Kramer, 1988). The listener is immersed in a sonic environment where dynamic and sound-shape features (attack, decay, sustain, release) are regular, allowing listeners to focus on the subtle details and nuances of sound. It is evident that in this case the composer does not look for the unexpected, there is no functionality in the temporal organisation of sonic events, but rather a kind of plastic presence. A piece such as *Ilmenemismuoto/Appearanceform* by Pan Sonic (Vainio & Väisänen, 2004) is composed of discrete blocks of sound interposed with silence and quiet episodes. The piece moves from extremely noisy sounds to microscopic interventions in the high frequency range. At first listening, one may be tempted to adjust the volume to temper these dynamic contrasts. The very slow decay that starts at 01’08” serves to prepare the listener for the delicate sounds that follow. There is a clear intention to challenge the listener’s perception with a drastic range of sonic material, and therefore a very good listening environment is essential: the lack of this would completely compromise the appreciation of the piece. Sound is treated as if it were a plastic substance, it is stretched, compressed, and dilated; extreme dynamic contrasts enhance the effect of sound having a physical consistency. In general, these pieces try to push the listener towards another appraisal: this musical material is not representational of something but is *formally* sonic.

The general idea behind the work of these composers is to encourage the audience to find new and different ways to approach the musical experience, which may involve provoking particular listening attitudes (Gérard Grisey, Francisco López, Pauline Oliveros) or exploiting the resonance of a physical space (Iannis Xenakis, Alvin Lucier), thus moving towards immersive phenomenal experiences. Many composers working in this area explicitly reject the idea of music as expressing or representing a specific meaning, emotion, or concept, preferring instead an idea of music as stimulating an aural experience that each listener engages with in their own way (López, 2004; Oliveros, 2005; Gross & Pitts, 2016). We, as theorists and analysts, should approach these pieces not only as challenges to existing paradigms but they should also be understood as putting forward a new way of thinking about music and perception.

The cognitive models employed in empirical musicology (Brattico et al., 2013; Hargreaves & North, 2010; Juslin, 2013) function according to certain traditional dimensions, such as musical meaning, aesthetic judgment, emotion (expressed and induced), and episodic memory, that vary according to the cultural context and the musical characteristics. These traditional theories of music interpretation have been enriched through an expanded understanding of the musical experience as also encompassing free associations, phenomenological perspectives, and intuitions, and as being affected by dynamic and temporal elements (Deliège, 1989; Imberty, 2005). This is of particular significance when considering sound-based music. The distinction between the representation of emotions versus the arousal of emotions (Juslin, 2013) starts to break down when examining music typical of today’s experimental scene. From the limited number of perceptual studies that deal with today’s contemporary and experimental music, there emerges the idea that listening has more to do with feelings than with codified emotions, and that “contemporary art […] [has] placed more emphasis on phenomenal qualities than emotions” (Kendall, 2014, p. 200).

I am not suggesting that the emotional response to sound-based music does not exist, but rather that the best way to understand how we experience this music is phenomenological, that is by looking at the mechanisms of perception we use in relation to sounds from the wider physical context. It is not by chance that most studies on the perception of current styles of experimental music draw on phenomenological perspectives and Gestalt theories (Koffka, 1935/1999; Merleau-Ponty, 1945/2003; Varela et al., 1991), elaborating on several disciplines within the cognitive sciences, the philosophy of art, and ecological theory (Solomos, 2023; Clarke, 2005; Gibson, 1979). Starting from these viewpoints, scholars –mostly in sound art– claim that an intrinsic capacity of sound objects is to allow sound to be perceived “in itself”. In this way, these studies forgo any discussion of the emotional effect of music. Rather, in going beyond Schaeffer’s reduced listening, these authors propose the possibility of sounds holding a value that is inherent to sound itself (e.g. “affordance”) (Kozak, 2020; Krueger, 2014; Menin & Schiavio, 2012; Reybrouck, 2012; Windsor & de Bézenac, 2012) or that is part of poietic and aesthetic spheres and which “refers to the world beyond the musical work” (Demers, 2010, p. 36) (e.g. “Intention/Reception” project (Landy, 2007)).

A possible contradiction may arise. On the one hand, it appears that sound-based music bypasses any dialectical and representational discourse and so points directly to a sensorial/phenomenological appreciation, something that apparently come close to a quasi-objective physical realm. On the other hand, the listener seems to be free to engage with it in multiple subjective ways (e.g., immersive, conceptual). This ambiguity is apparent simply because sound-based music can be appreciated in diverse fashions, as is true for other genres of music; a Beethoven string quartet can be listened to by focusing on the sonic impact of its musical episodes instead of its tonal structures or emotional effect. By its preference for the nature of sound to the detriment of its function, sound-based music allows this focus on sonic material to occur more easily. This music, as we will see, is defined by a series of sonic patterns organized by laws which resemble the natural schemes we encounter in the world, such as figure-background, force-counterforce, near-far, cyclic repetition, superimposition, and blockage. These schemes are constitutive of all music (Larson, 2012; Lerdahl, 2001), but in the case of sound-based music they are central in the organization of sound patterns and have the potential to open up towards a large variety of cognitive responses. This is to say that our perceptual engagement with sound-based music seems to be related to a connection to the world in an everyday sense, in contrast with other kinds of music where knowledge of specific musical languages and cultural codes is involved.

## The characteristics of sound-based music: the perceptual grammar

In analyzing pieces from this cross-genre set of music practices, I employed general categories outlining morphologies and evolutions through which musical episodes may be described and compared (Wanke, 2015; 2017). Several methods (Smalley, 1997; Giomi & Ligabue, 1998; Roy, 2003; Emmerson & Landy, 2016; Delalande, 2013) allow for a sort of aural and morphological analysis which favours the identification of correspondences between the pieces in the use of spectro-temporal sonic details (e.g. *glissandi*, microtonality) and the arrangement of musical figures (e.g. types of repetition or figure-ground configurations) both on the micro- and macro-scale. Some musical attributes were defined that address both low-level essential characteristics (morphologies and structural forms) and high-level more complex arrangements (global configurations and larger temporal constructions) that often involve combinations of low-level attributes (Table 1).

**Table 1:** Sonic characteristics and typologies of the Perceptual Grammar (Wanke, 2021; 2015).


TYPES	ATTRIBUTES/CHARACTERISTICS

Morphologies	• The use of an expanded spectrum

	• Interactions between neighboring frequencies (*e.g.*, microtonality, binaural beats)

	• Systematic frequency-based glides (*glissando*)

	• Static masses of sound (*e.g.*, sustained tone-based aggregates, drones)

Evolutions	• Metric structures (*e.g.*, rhythm, repetitive clusters)

	• Dynamic and Timbric Contrasts

Effects	• Hypnotic Reiterations

	• Auditory challenges (*e.g.*, distortions, illusory effects, psychoacoustic phenomena).

	• Macro/micro constructions.

Arrangements	• A plastic and sculptural arrangement of sound

	• Restricted number of elements conceived globally

	• Limited dialectic among elements


The attributes taken as a whole account for an analytical field of similarities between pieces that come from a diverse panorama of experimental music but that crucially share a common perspective on sound (Wanke, 2021). This field can be divided into low- and high-level attributes covering morphologies, unfolding structures, their effects and arrangements. These attributes, due to their nature, are often used in combination: for instance, glitch-electronic music usually exhibits repetitive clusters within rhythmic frameworks, while the repetition of musical units in longer compositions from the repertoire of contemporary classical and electroacoustic music can at times be associated with non-rhythmic hypnotic reiterations or more complex structures. This analytic field is made of interdependences and hierarchies in such a way that these attributes form a sort of *perceptual grammar* incorporating internal rules and synergies. It is not merely a lexicon of sound types –which may recall the idea of sound morphology in *musique concrète* (Schaeffer, 1966)– rather it is a collection of characteristics interrelated within a sort of primitive syntactic structure. The concept of perceptual grammar parallels Kanisza’s visual grammar (1979), insofar as this perceptual grammar embodies Gestalt principles of proximity and similarity and embraces a series of essential (identity, difference, motion, and stasis), structured (ascending/descending, figure/background), and dynamic or kinaesthetic (tension, linearity, amplitude, projection) patterns (Tenney, 1988; Sheets-Johnstone, 1999; Johnson, 1987). At the same time, the perceptual grammar fits within a broader panorama of cognitive grammars (Langacker, 1987). In this context, a cognitive grammar is a perceptually rooted natural grammar (Petitot, 2011, p. 25) which does not function as an autonomous level of representation but rather is a bridging domain between physical and symbolic levels endowed with geometric features (dimensions, distances, continuous/discrete topologies, boundaries, etc.) and “intensive” and “extensive” magnitudes. The perceptual grammar consists of a series of interrelated features that concern qualitative and quantitative dimensions, arrangements and evolutions of sound patterns within a dynamic and spectrotemporal domain.

We can find examples of sound patterns and their organization within the perceptual grammar of pieces spanning post-spectralism, glitch-electronica, electroacoustic music and other practices in sound art. If we consider, for instance, the short track entitled *If Anywhere Was Here He Would Know Where We Are* (2010) by the London-based electronic musician Raime and the piece *Dulle Griet* (2009) by the Italian composer Giovanni Verrando, we can find good examples of sound patterns within kinds of sculptural arrangements. Sound events in these pieces are organised and perceived based on spectral and dynamic qualities in order to create different planes or dimensions of perception. Sounds emerge, appear, and display themselves within a sonic/perceptual domain as they occupy a particular spaciousness and hold a sort of tactile and plastic presence. In Raime’s piece, the music unfolds by describing a sort of climax-anticlimax structure in which all elements work as constituents of a larger sonic idea. Verrando conceives each sonic scene through the total accumulated sound matter, he explores the interrelation between harmonic and inharmonic sonic forms, using these elements in opposition and alternating between acoustic and electric sound sources. In identifying the experiential framework as a sort of spectral canvas, listeners spontaneously segregate what element is positioned in front or back, and what is repeated or transposed.

**Figure 1:**
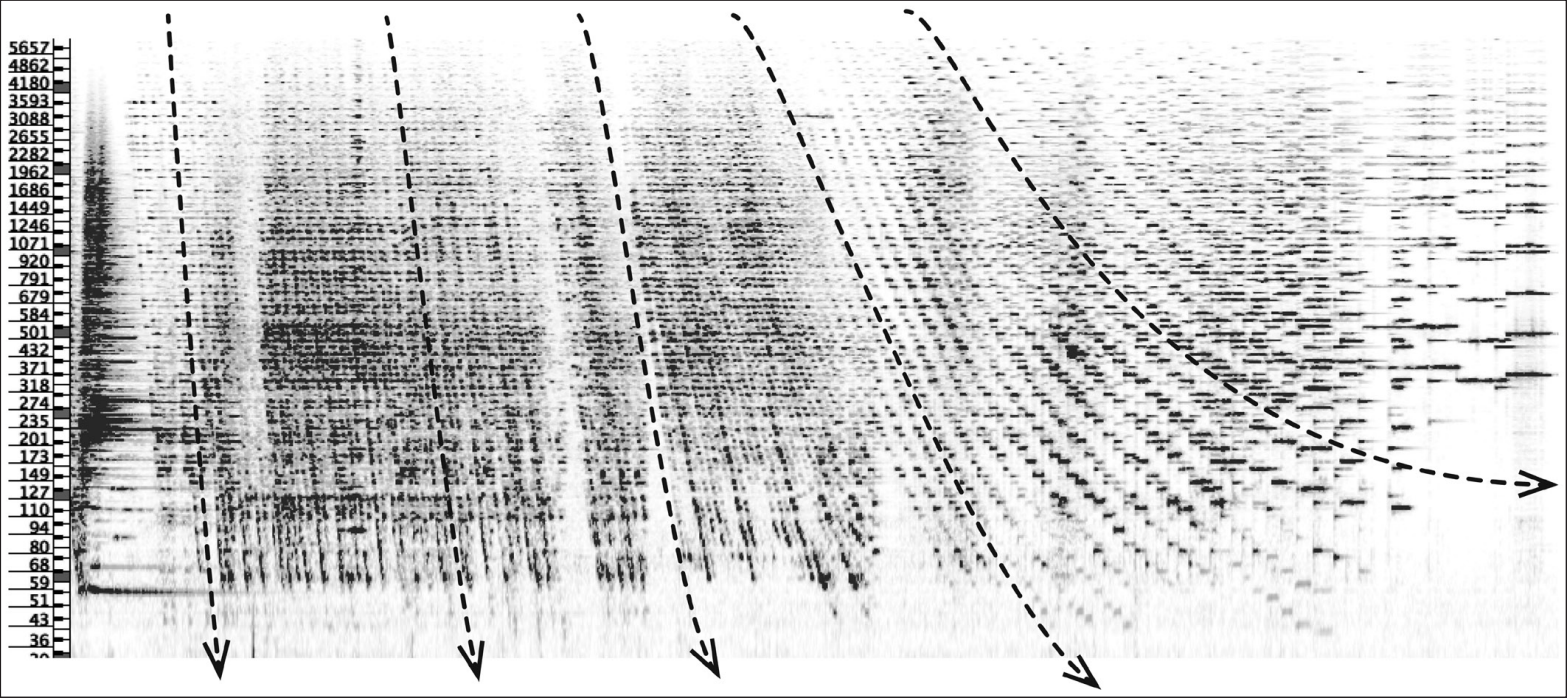
Spectrogram [30 – 5700 Hz/31’11” – 35’21”/bars 343 – 413] of Haas’ *in vain* (2000).

The orchestral piece *in vain* by the Austrian composer Georg Friedrich Haas (2000) provides a good example of the combined use of several attributes of perceptual grammar.^3^ The piece, considered as a whole, is based on a sort of primordial opposition between elements, whether dynamic, temporal, rhythmic, or relating to tuning systems (i.e. natural *vs*. tempered). The piece is characterised by symmetries, a frequent use of repetition to create larger structures, and a sequential (and progressive) modulation of sonic constructions. The episode which opens and closes the piece (the opening figure being mirrored at the end) consists of the reiteration of descending notes evoking a sort of large-scale falling spiral that apparently ascends due to the continuous re-exposition of higher lines, thus recalling the never-ending development of some acoustic illusions such as the Shepard tone (Figure 1). The descending lines which begin the figure are weaved together into a dense sequence of notes and their fast repetition generates a sort of sustained and oscillating effect of suspended tritones.^4^ From a fast succession of short tones within a steep descending configuration, Haas progressively dilates the entry points of new notes and their durations, modifying the sequential overlapping of every descending figure (Figure 1, dashed arrows).

Even if some pieces, such as *in vain*, can be appreciated and experienced in multiple ways (narrative, symbolic, sensorial), there is a more general and overriding impulse in sound-based music that pushes the listener toward the sonic experience itself. Sound can be identifiable (e.g. instrumental sounds) and some sonic patterns may recall traditional harmonic progressions (e.g. a sequence of ascending clusters based on the progression of different fundamental notes^5^), but the sonic events themselves are part of a unified conception of sound. This music does not progress according to a functional design (see Table 1, Arrangements), but rather it focuses on the presentation of aural elements while minimizing their articulation: the music is characterized not by a narrative relationship between elements but by a grammar that governs the total accumulated sound material. In the case of the pieces described here, the focus on sound as a material is also brought about by the lack of everyday sounds: while this is a characteristic element of *musique concrète*, the music considered here does not include recognizable real-world sounds and is centred on the nature of sound itself thus limiting semantic interpretation (style, genres, instrumentation) and factual listening (production, contexts). There is a subtle, but crucial, difference in the aesthetic (and the perceptual potential) of pieces such as *De Natura Sonorum* (1975) by Parmegiani or *Hétérozygote* (1964) by Luc Ferrari, which I do not consider as sound-based music, and other pieces of the same period such as Giacinto Scelsi’s *String Quartet n.4* (1964) or György Ligeti’s *Lontano* (1967), which I do categorise as sound-based. Parmegiani and Ferrari use both manipulated real-world sounds and electronic sounds in an interplay of sonic events which constitute realistic (or seemingly realistic) scenarios. In Ligeti’s and Scelsi’s pieces, on the other hand, sounds are parts of a global sonic texture where the interest lies not in its articulation but rather in its nature.

In sum, the perceptual grammar characterizes the organisation of sound patterns within an aesthetic-perceptual domain through which we experience this kind of music. This grammar is constituted by attributes which typify shapes, evolutions, effects, and arrangements of sound patterns, and it is governed by dependencies and hierarchies which follow Gestalt and kinaesthetic principles. The pieces embodying this perceptual grammar are made of formal structures that are *sonic* and are experienced as they are (as opposed to representative of something else). This notion of the formal as topological and geometrical but not symbolic nor logical (Petitot, 2011, p. 50) is an aspect that may lead to a cognitive turn in how the listening experience of this music is thought of.

## Morphodynamic theory applied to sound-based music

While the appreciation of contemporary art music still involves general mechanisms that are dependent on familiarity and background (Grebosz-Haring & Weichbold, 2020; Menger, 2014; Bourdieu, 1979), I propose here that this kind of music is particularly well-suited to being studied at a perceptual and cognitive level. This investigation goes beyond the field of music listening and involves a broad field of disciplines spanning cognitive psychology, neuroscience and philosophy of mind.

Looking at the aesthetic experience of sounds allows a focus on how we interact with and interpret acoustic stimuli outside the reach of an everyday context and any evolutive theory: a melody or a *glissando* is a concatenated (or continuous) series of acoustic stimuli which unfolds within a perceptual domain that is independent from the external world and its phenomenal information (cause, directionality, position, external movement…). As Dowling cleverly points out:

[during a performance of a sonata for violin and piano], [t]he piano as a sound source is located in physical space, along with the violin, the rattling of page turns, coughs in the audience, etc. The melody as a sound object is, in contrast, located in a kind of “virtual space” of pitch and time […]. Kubovy and Van Valkenbug […] pointing out that the closest auditory analog of shape perception in vision is the perception of melodic contours traced in pitch across time (2010, p. 231).

The experience of the *world of sounds*, to use an expression of Roger Scruton (1997), involves cognitive processes typical of abstract topics, such as mathematics, geometry, and Gestalt theory. However, the cognitive domain inhabited by a melody is not one of abstract logic, as with formal languages (formal logic, formal semantics, categorical grammars, etc), but rather is strictly connected to the sensorial and external physical levels.

Several studies discuss how musical patterns, by belonging to this sort of virtual space, are experienced as musical motions and forces (Larson, 2012; Larson & VanHandel, 2005; Lerdahl, 2001; Brower, 2000; 1997), but there is an important difference here between pitch-based and frequency-based movements. Most of the studies discuss music in general terms and do not differentiate between tonal and atonal music, or between 12-tone equal temperament (12-TET) and microtonal systems, or more generally between note-based and sound-based music. These studies typically state that musical motion between notes (i.e. with stable pitch) leads to some sort of perceived effect. Snyder, for instance, reports the sensation of motion provided by a sequence of notes as an *illusion* (2001), whereas Johnson discusses the experience of the musical motion “as [being as] real as temporal motion” (2008, p. 255). We experience a fast or slow sequence of notes as a temporal dimension of music, but what is being debated here is the spectrotemporal domain. Melodies are in fact musical patterns within a multidimensional *space* of frequency, time, and intensity. While time is a continuous dimension, in the sonic patterns of the most common genres of music, frequency movements are *formally* “quantized”, i.e. 12-TET. This concern is well expressed by Zuckerkandl:

motion is the process that conveys the thing from here to there, in a continuous and never suspended traversal of the interval. If it stops anywhere, the motion is instantly abolished. But in a melody we have nothing but this, nothing but stops, a stringing together of static tones, and, between tone and tone, no connection, no transition, no filling up of intervals, nothing. It is the exact opposite of motion (1969, p. 83).

The difference between a descending sequence of notes and a *glissando* is evident here. If a motion is felt by listeners with a pitch-based stepwise sequence, this effect would be even stronger, and I would say *real*, in the case of sound movements with continuously shifting frequencies. This is why it is important to be precise on what type of musical style is being considered. The sound patterns of sound-based music often consist of continuous movements in the overall spectrotemporal domain and can even present psychoacoustic effects: when listeners experience *glissandi*, binaural beats, and microtonal cross-fading transitions between dense sonic clusters, they are experiencing *real* movements in this perceptual domain.

Pieces by figures such as Giacinto Scelsi, György Ligeti, Eliane Radigue, Emptyset, and Mika Vainio explore sonic features as essential aesthetic elements: these works, in contrast with more traditional styles of music, are made of sonic structures whose nature remains at the foundation of their aesthetic appearance. The formal level of this music is not symbolic nor functional but rather topological and geometrical.

Morphodynamic theory proposes a connectionist model of cognition in which the structures and patterns of the physical external world are made explicit to us through their morphodynamics. It identifies in the visual geometry of perceptual scenes abstract invariants that can be reformatted as syntactic constituent-structures. Initiated by René Thom in the 60s, morphodynamic theory (Thom, 1983; Petitot, 1985; 2011) has drawn on mathematical models of singularities and dynamical systems and applied them to structural phonetics and semiotics, and to categorical and visual perception (Laeng, 2013; Petitot, 2011; Kosslyn et al., 2007; Hampe, 2008; Marr, 1982; Lakoff & Johnson, 1980). In the cognitive sciences (Petitot, 2011), the morphodynamic model works on a subsymbolic cognitive level connecting the external physical level to the symbolic one. Abstract structures of meaning are, in the morphodynamic perspective, *natural* structures that can no longer be seen as formal assemblages of symbolic elements connected by means of formal relations. They are natural emerging Gestalts, self-organised and dynamically regulated wholes, experienced as forms of patterns.

When applied to the cognitive mechanisms that are at the heart of the perceptual experience of this music, morphodynamic theory sheds light on the link between the sonic structures perceived by listeners and the evoked mental images. Morphodynamic theory comes into play in understanding how external sound patterns correspond to the experience of internal geometries in motion. In the case of auditory perception and in particular of music cognition, some works (Reybrouck, 2013; Godøy, 1997) put forward the idea of a shape-paradigm where sound patterns can evoke mental representations in the form of geometries and shapes. In preserving the frequency-time nature of sound, “geometry must be the primary element in representation, primordial to other more abstract, symbolic and/or numerical representations. […] Also, geometric representations of qualities as shapes have the advantage of being more in accordance with the approximate nature of perception and cognition, […]” (Godøy, 1997, p. 94). In order to describe how morphodynamic theory can be used here, I find appropriate, for the definition of these cognitive patterns, the notion of “image schemata”. Image schemata are abstract and recurring mental images which, by having an inherent spatial structure and a kinaesthetic character, are able to bridge our perception to the external world within an embodied experience (Johnson, 1987).

Morphodynamic theory could be straightforwardly applied to music seen as organized sound forms. This translation is even more efficacious if we consider sound-based music. On the one hand, the traditional symbolic paradigm states that mental representations begin as internal formal structures: the cognitive system provides its internal symbols with meaning. They are symbolic, meaning they relate to internal mechanisms of thought. By following this formalist stance, these mental representations are then not directly associated with the rules governing the physical world. The latter, conceived in a physicalist sense, is a priori without relevant meaning for the cognitive system. Petitot talks about an “ontological gap” that separates symbolic and physical levels in traditional cognitive theories (2011, p. 51). On the other hand, the morphodynamic model is a connectionist model because it tends to address this gap between symbolic and physical levels (Smolensky, 1988). A morphodynamic level, in fact, (i) has a physical origin: morphological information is preserved through transduction (Marr, 1982), (ii) is geometric-topological, and (iii) is not symbolic nor logical but continues to be formal.

As previously stated, the music considered here displays essential sound patterns arranged and constructed following Gestalt principles. The nature of these patterns is tied to the early stage of our perception: the auditory Gestalten formation and the functioning of working memory (Koelsch & Siebel, 2005; Wanke & Santarcangelo, 2021). Listeners grasp these patterns within a perceptual domain as profiles and shapes that unfold, expand, and collapse, and articulate their perception of sound in terms of lines, planes, layers, and degrees of density (Wanke, 2019; Godøy, 1997). This domain is an extent where the sound patterns of the outer world are transduced internally, from physical to electrical energy, through the processing of our perception in which the “cochlea constructs sound images with dimensions that correspond to time and frequency domains” (Griffiths & Warren, 2004, p. 888). Segregation based on differences in spectral content occurs via a tonotopic organization from the cochlea throughout the auditory pathway up to and including the auditory cortex (Ruggles et al., 2018; Bregman, 1990). During this first stage, the idea of a relationship between external patterns and neurological response has been proposed in the form of an isomorphism that is functional rather than physical (Fingelkurts et al., 2010): an internal neuronal process that corresponds to the experience of an external stimulus of a “circle”, for instance, does not involve neurons arranged in the form of a circle. This means that this relationship is not of the first order but rather is functional: neurological and sensory systems consist of different realizations of the same kind of process. If the mindless brain activity is isomorphically related to the spectrotemporal features of the acoustic stimulus, it seems plausible that the successive cognitive steps at a higher-level should keep a trace of these features (DeLanda, 2022). Morphodynamic assumptions depend exactly on the fact that the qualitative and quantitative discontinuities of both sound patterns and image schemata are transmitted from substrata to substrata.

If musical episodes containing oppositions of sonic aggregates, transient appearances of fragile overtones, or continuous descending lines are processed as energy-forms evoking mental images such as empty or solid blocks, oscillating lines, or descending profiles (Wanke, 2019), it seems plausible to think that a perceived *glissando*, i.e., an effective continuous motion in the confines of frequency, time and intensity, could originate a functionally coherent set of responses which preserve the constitutive elements of the *glissando* itself, such as those of wholeness, continuity, directionality, movement, and progressive change.

This connectionist proposal may find correspondences in non-representational theories in the cognitive sciences, namely ecological psychology (Gibson, 1979) and enactivism (Varela et al., 1991). Both of these theories reject the idea that cognition is based on computational operations of internal mental representations and propose instead a bridge to the environment as a frame of references affording meaning (Gibson) or as a context in which to act (Varela). Applied to auditory cognition, and in particular to music listening, these two stances have been represented by two visions: on the one hand, some studies suggest the possibility of sounds offering an “affordance”, a value that is inherent to sound within a subject-object perceptual framework (Krueger, 2014; Menin & Schiavio, 2012; Windsor & de Bézenac, 2012); on the other hand, enactivism in music practices emerges within the broad notion of embodied cognition (Van der Schyff et al., 2022; Reybrouck, 2020; Leman, 2008). The morphodynamic understanding of the sound patterns discussed here places itself in between these two visions: it is crucially grounded in a specific type of musical sound and its characteristics (i.e. perceptual grammar), and it asserts the potential for a cognitive embodiment. While both visions consider music in general terms and do not develop a musicological distinction between genres and styles, the argument brought up in this paper is grounded in the specificity of certain music genres, which –it is argued– may favour a particular listening experience.

## The virtual embodiment of sound-based music

While more conventional genres of music (e.g., classical, popular and world music) rely on a series of stylistic schemes which call for culturally-guided interpretations, certain genres within sound-based music develop musical episodes which are better understood through their sonic presence than through any dialectical relation to them. They elicit image schemata that relate more to our being in the physical world (e.g., figure-background, near-far, superimposition, compulsion, blockage) than to any kind of representational musical construction. These musical episodes do not refer to any non-sonic information but rather to themselves as sonic constituents of the aesthetic experience. Sound in these pieces emerges as having a particular kind of material presence (Wanke, 2021).

The listener experiences the sound patterns of this music as stimuli that do not point to anything specific in the world but convey movements defined in terms of tensions, paths, amplitudes, and projections: the qualitative structures of all movements (Sheets-Johnstone, 1999; Johnson, 2008). Given this, listening requires a reconsideration of the listener’s embodied movements in the world and their experiential knowledge. The knowledge collected through embodied movements allows the individual to experience the qualities of things, spaces and forceful exertions.

We [can] experience linear versus nonlinear paths of motion, whereby we develop our understanding of trajectories. We feel various degrees of exertion and force, and we thus learn what level of exertion is appropriate for moving ourselves from one place to another and for moving objects of various weights. […] Movement is thus one of the principal ways by which we learn the meaning of things and acquire our ever-growing sense of what our world is like (Johnson, 2008, p. 21).

A descending *glissando* acts immediately on our body giving us, for instance, a sense of falling; a sequence of distorted sound pulses evokes motion through a series of solid obstacles. This is due to the fact that these sound patterns are made of physical forces that actually impact us, as listeners; they activate the basic structures of sensorimotor experience by which we find a world that we can act within. The sense of falling or the presence of thickened objects is nothing more than our experiential response to this impulse. Our primal associations are embodied as they meet a demand for a link to our bodily experience. These kinds of “geometries in motion” convey information about the world we inhabit, but they are not from the real world, rather they are from the phenomenological world of sounds. This point defines the kind of embodiment this music can provide.

Generally speaking, listening to music can be the occasion of one of the most intense disembodied experiences: when we are at home, seated or even reclined in our preferred sofa, listening to music with closed eyes, we can almost lose the sense of our body. Embodiment with music is, on the other hand, straightforwardly related to the bodily movements with the music. An embodied listening activity could also be represented by an attentive walk within a forest where sound cognition is largely based on our natural processes of external recognition: source position (directionality) and cause identification. Here, I discuss an embodiment with sound that is not associated with a corporeal response (dancing, tapping…) but rather is limited to its immanent potential and gives rise to a broad set of high-level cognitive associations. This embodiment is somehow virtual because the images evoked by these sound patterns are mental patterns originating from the auditory system, they reveal aspects of the physical characteristics of the incoming stimuli but they are not representations in the traditional sense and this is why the term “mental representation” is not the best choice here. These image schemata are in fact not *about* some external content, they do not represent actual objects or events, rather they are patterns of our meaningful experience.

This apparent conflict –the lack of a link to the external source of sound vs. the connection to the experiential world through the presence of essential forces in sound– is the central point of the virtual embodiment seen as a *potential* for embodiment. The listener apprehends not just a sonic material but also a potential physical energy-form. The experience of sound patterns pushes beyond the merely sonic towards a response to these energies. To listen to this music is to search within a personal sense of physicality and relation of perceiving body to the world (i.e. Johnson’s *image schema*), a relation made of tensions, motions, contrasts, and recognitions. It is worth recapitulating here three crucial aspects which typify sound-based music:

- First, its sound patterns are appreciated at an aesthetic level, they are presented to the listeners as music rather than as general acoustic stimuli. The approach taken to these pieces tends to limit any factual listening. This is to say that, for instance, a continuous sound that begins quietly and increases in dynamic intensity, is not understood as a sound source approaching us, nor a distorted pulsation as an explosive event.
- Second, its perceptual grammar does not call to any representational or stylistic level. In this way, these pieces potentially minimize the culturally-guided elaboration of concepts and beliefs.
- Third, the nature of the perceptual grammar is grounded in Gestalt and kinaesthetic schemes. The movements that this music portrays are the same as those that we encounter in our experience of the world.

The combination of these aspects leads to the idea that, compared to other auditory stimuli, the sound configurations of this music are in fact unique: they limit the semantic listening (factual, culturally-guided), they are prone to an aesthetic appreciation but open to an embodied engagement.

## Methods

### Listening Survey

I carried out two listening questionnaires (Wanke, 2022) proposing potential correspondences between musical excerpts and visual drawings in order to (i) observe how certain principles of morphodynamic theory function in practice (mainly the idea of the preservation of tonotopic distribution (frequency-time) from the acoustic stimuli up to its mental representation) and (ii) explore the relation between sound patterns and image schemata based on the participants’ responses. The main objective was to identify any tendencies in how the participants approached the visual representation of this music relating to their background and to the sound types I had previously analyzed.

The surveys were carried out through a web-based platform (i.e. via personal and academic contacts, and social networks). The beginning section aims to establish a profile of the listener (age, link to music, musical preferences, and familiarity with the audio samples). Following this, the perceptual evaluation consists of listening to nine audio samples: for each sample, participants are asked to evaluate the degree of correspondence with a pair drawings and to include personal comments and remarks. The final section includes questions about the listener’s opinion of the audio samples, the drawings and the questionnaire itself in order to obtain insights concerning the level of interest of the participant for the present investigation and possible suggestions for improvement.

### Participants

Sixty-seven participants took part in the experiment (43 male, 21 female, 3 do not specify, age: M = 29.0 years, SD = 1.13). A majority (79%, 36 male, 15 female, 2 do not specify) reported a prior professional (or semi-professional) link to music through being either students of music or musicology, musicians, or scholars, and only 14 subjects (7 male, 6 female, 1 does not specify) declared to be musically “untrained”.

### Audio Samples

Audio extracts (9, time range: 15-45 s) were selected based upon previous studies (Wanke 2021; 2019). The compositions belong to specific styles of experimental music, namely post-spectralism (Haas), electroacoustic and mixed music (Momi), and electronic-glitch music (Demidike Stare, Pan Sonic, Emptyset).^6^ Haas’ pieces (*String Quartet n.2*, *in vain*) are traditionally written compositions for acoustic instruments that focus on microtonal techniques derived from tuning oppositions (i.e., 12-TET system *vs*. just intonation). Momi’s work, on the other hand, falls within the broader category of electroacoustic music, however his compositions reflect contemporary classical aesthetic traits with a complex use of real-time electronic manipulation. The excerpts by Demdike Stare, Emptyset and Pan Sonic belong to the large variety of music practices within glitch-electronica and consist of electronic sounds with no presence of natural sounds. The selection of extracts was made while trying to avoid misrepresenting the nature of each piece, but naturally each extract is a short focused musical episode lasting between 15 and 45 seconds crucially containing only one type of event (single or repeated).

### Drawings

Drawings were prepared to represent two general relations between things: static and dynamic (Petitot, 2011, p. 172). In the case of music, these characteristics may correspond to the linear representation of succession of sounds, on the one hand, and the static portrayal of the auditory scene on the other. They reflect spectrotemporal profiles based on the qualitative assumption that the vertical dimension represents the tonotopic discrimination of the frequency domain (Cox, 2016; Küssner, 2018).

## Results

The linear spectrotemporal representation of sound samples is generally preferred. To explain this, one should first consider that when sound patterns are of types for which no causal actions could be identified (as for the pieces considered here), these sound patterns can elicit movements that reflect spectrotemporal contours (Caramiaux et al., 2014). Second, a perceived movement helps conceptualize the passing of time: there is always a *before*, a *during* and an *after* (Sheets-Johnstone, 1999), and our common experience of musical motion depends upon the habit of representing the properties of time in space. We express duration in terms of extension and so the sequence of events takes on the appearance of a continuous line (Bergson, 1910/2008). Finally, spectrotemporal representation is usually preferred by experienced listeners who are used to thinking of a visual rendering of music in terms of a spectrogram and a waveform, as expressed by one of the participants:

in some cases, both figures seemed appropriate. The choice was influenced by some biases, two most of all, both linked to the fact that the relationship between music and drawing lies in notation: the habit to ‘read’ time from left to right, and that to read pitch from low/bottom to high/upper part of the image. In few cases, timbre and texture played a major role in perceptual matching.

**Figure 2:**
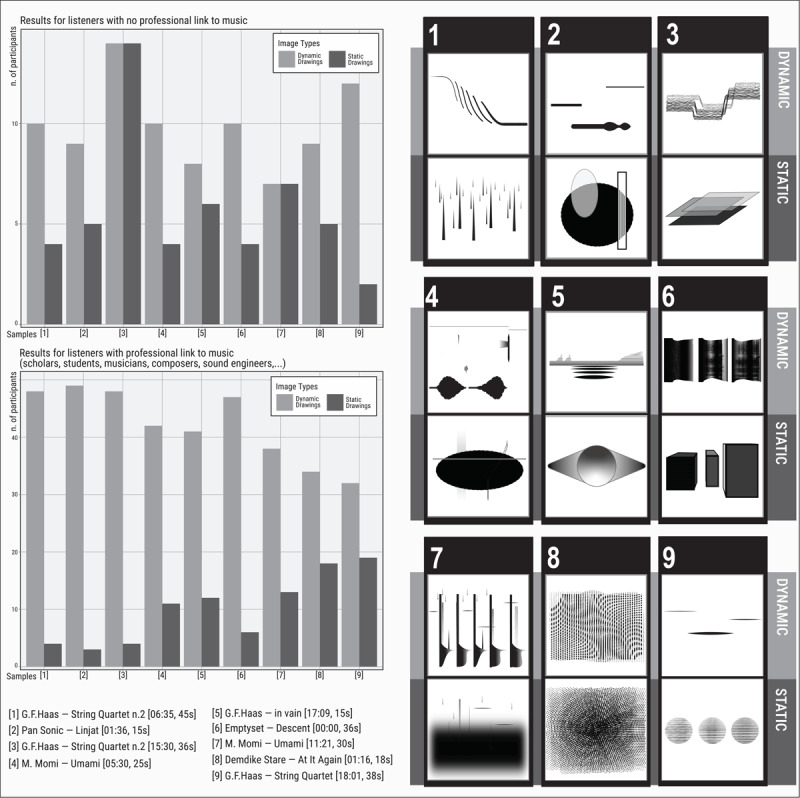
Left side: schematic representation of the results sorted by participants’ musical training. Bar graphs where pale grey bars represent “dynamic” drawings (linear and spectrotemporal evolution) and dark grey bars represent “static” drawings (Static portrait). Right side: the nine pairs of drawings.

Besides an overall preference for the dynamic linear visualizations of sound patterns, an analysis of the differences in response between trained and untrained participants reveals that trained participants tend to have a higher preference for dynamic linear visualization than untrained ones (Figure 2).^7^

As mentioned above, daily use of music editing and production software or simply regular contact with musical scores leads trained participants to be familiar with the linear representation of sounds within a frequency/time domain. Some signs in the dynamic drawings may recall those used in various musical contexts (acousmatic or graphic scores), therefore someone familiar with these contexts might see those signs as more accurate and detailed than the static representation, which may appear as a simple interpretation of sounds in visual form. However, linear visualization of music evolution is so common in our everyday life (*e.g*. the waveform representation of voice messages in applications on our mobiles phones) that the difference between trained and untrained listeners does not appear too marked. One could argue conversely that the static representation may require the capacity to conceptualize the entire episode as a unique event and thus should be preferred by experienced listeners. Even if these samples are quite short, thereby facilitating the static representation, the general tendency, in the context of this listening survey, is to listen and collect the chronometric sequence of sonic events.

Overall, the response of the participants appears to be consistent and does not vary too much according to age, the time taken to complete the survey, or musical training. There is a substantial agreement among participants in expressing a preference for linear representations of sound patterns.

### Shortcomings

The survey tries to balance the degree of freedom given to participants: it uses set choices (i.e. the pair of drawings) but also provides listeners with space for their own comments. Offering more types of drawings to choose from or letting participants draw by themselves could lead to a richer account of the musical experience, but at the same time this could lead to a set of data that is difficult to analyse. Furthermore, having participants draw by themselves favours a post-experiential description of listening (e.g., elaborating figurative representations), while the survey aims to encourage listeners to focus on the immanent auditory experience of sound patterns themselves. The set of well-controlled tasks helps to get a more solid and coherent set of data. Due to the characteristics of the music samples, which come from uncommon and, somehow, challenging music pieces, a survey containing given options is generally preferred by participants. The drawings, in fact, have a coherent and clear aesthetic (non-figurative and with a linear/static opposition), which helped participants focus on the cross-modal task. Finally, the Western cultural bias of interpreting temporal evolution as a line from left to right and frequency vertically is an evident limitation but one that is uniformly accepted in this type of study (Küssner, 2018).

## Discussion

**Figure 3:**
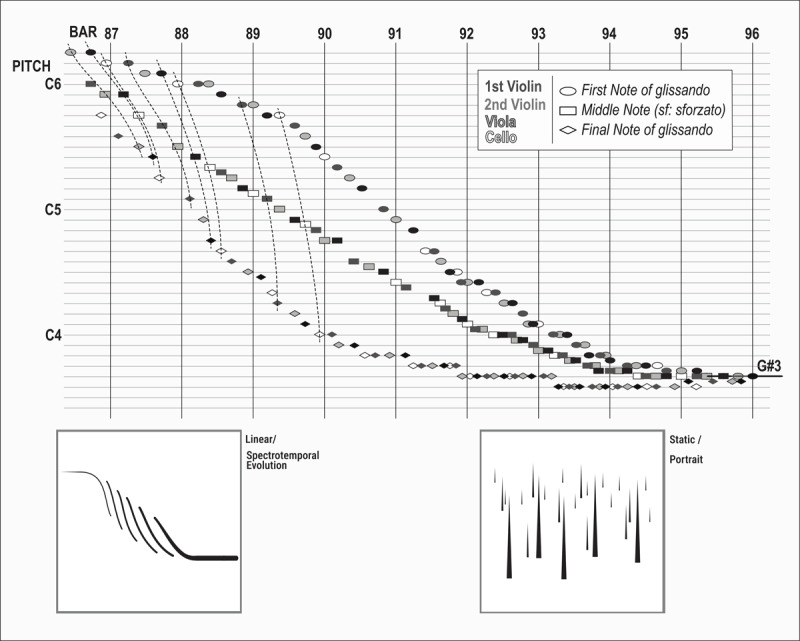
Top: pitch-based representation of interlaced glissandi of Haas’ *String Quartet nº2*, bars 87 – 96 [06’45” – 07’25”]; Bottom: corresponding drawings.

Sample #1 of the survey comes from the *String Quartet nº2* (1998) by Haas and provides an interesting first example: Haas constructs an episode using repetitive *glissandi* played with the same dynamic structure (*i.e*. a middle note in the *glissando* is played *sforzato*) to build a larger descending continuous cascade. This movement (from E-flat-6 to G-sharp-3, bars 87–96) is generated by the interweaving figures of the four instruments. It starts vertically (steep *glissandi*) and ends horizontally (i.e. sustained tones moving within a microtonal range of frequencies): Figure 3, at the top, displays a schematic representation of the episode where first, middle and ending notes of each *glissando* are plotted for the four instruments (together with the corresponding drawings from the survey, dynamic and static, at the bottom of Figure). In terms of pitch construction, the starting and ending notes of each glissando move down chromatically in semi-tone steps (except at the end of episode), while the middle notes describe a more gradual slope including quarter-tones. In this way, the central part of the episode has *glissandi* spanning larger ranges while at the beginning and at the end the ranges are smaller: there is an overall movement that progressively accelerates its descent and then at the end reduces its descent speed.

This sound pattern has the appearance more of a landing on the ground than of a fall into a chasm: the G-sharp-3 is gradually introduced from bar 92 as the ending note of the glissandi and then is progressively reaffirmed within a sequence that develops more in a horizontal sense than in the vertical sense of the central part (bars 89 – 91). This may be a reason why participants tend to prefer the linear drawing (where time could be understood as running linearly left to right), which better reflects this sense of horizontal ending.

**Figure 4:**
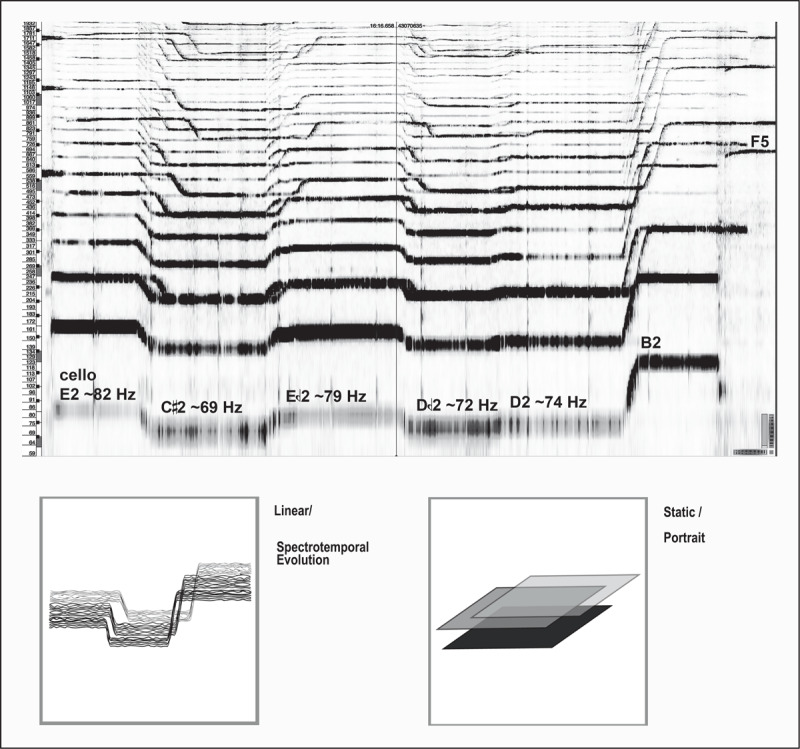
Top: spectrogram [59 – 2000 Hz/15’27” – 17’08”/bars 215 – 227] of Haas’ *String Quartet nº2*. Bottom: corresponding drawings.

From the same piece, sample #3 consists of a sonic flow (*e.g*. using fundamental pitches and their natural harmonic series) made of closely-spaced microtonal textures or delicate continuous lines of sound where each instrument moves in parallel (Figure 4). This episode has a horizontal development, all instruments play continuous single notes or simple chords and create a layered and continuous mass of sound, moving from one note to another through parallel (but not synchronous) *glissandi*.

Crucial here are the participants’ comments describing the listening experience:

Like paint or something dripping from the ceiling;

The sensation of something that slowly comes out from a static source;

Highly viscous substance oozing…

A certain idea of a viscosity, expressed by the participants in different ways, emerges from the sound patterns themselves: a sonic flow with several harmonic series that gradually change from one to another. By introducing a new fundamental note, the cello anticipates the new harmonic series while viola and violin lines remain *stuck* in the previous one. The perceptual engagement with this sequence seems then to revolve around an embodied experience connected to the world in an everyday sense. Johnson specifies that

we feel these qualitative dimensions. However, they are not just subjective qualities. It would be a mistake to subjectivize these experiences of qualities in motion, as if they were locked up within some private inner world of feelings. On the contrary, they are qualities of organism-environment interactions. […] They are qualities in the world as much as they are in us (2008, p. 25).

The association between a sequence of sustained tones and a viscous substance dripping down has a *cross-modal* character: listeners recognize in the music the same *abstract* pattern they found during a visual (or tactile) experience of such a substance. The “viscosity pattern” emerges then as a *common experiential pattern*. This ability to correlate structures from one perceptual domain with those from a different perceptual modality is a basic part of how we understand our world via bodily interactions (ibid. 2008, pp. 42–43). Moreover, participants’ remarks suggest that the very nature of this association is implicit: some people have a clear picture of the evoked vision, others account only for the most essential features (“…something that slowly comes out from a static source”).

In other cases, it is more difficult to extrapolate general observations: with the more complex samples (i.e. #4 #7, see Figure 2), the visual representation of the spectrotemporal sequence provides a set of detailed characteristics that static representations do not. However, for sample #4, linear evolution is preferred by both trained and untrained participants, whereas in the case of sample #7, untrained participants seem to prefer the static drawing where repeated pulses at bass frequencies partially masking the perception of the high frequency interventions– are represented in the drawing as a cloudy layer covering other signs. This visual representation is somehow simpler to deal with because it offers the possibility to untrained participants of identifying a predominant element that hides other sonic nuances. As observed in other studies (Wanke, 2021; 2019), the responses of unskilled participants appear to involve free associations while experienced listeners are more inclined to assume an analytic stance, being reluctant to open up to more spontaneous responses.

## Sound-based music as an example of a connectionist model

By employing a specific perceptual grammar, sound-based music offers a way to go beyond sound *through* sound and reach out to the level of wide-ranging embodied associations. Without recourse to extra-musical symbolism, this music, through its sonic forms, brings together participants in sound and yet manages to push their experiences towards a broader experiential and embodied level. While traditional music genres have the potential to *afford* something beyond sound that pertains to a conceptual cognitive level, the “affordances” of sound-based music convey forces, tensions and motions within sonorous vehicles: they are not references to any causality nor, at first glance, to any kind of concept of style, instrumentation, or compositional perspective. Sound-based music seems then to possess the characteristics which would allow a “cognitive turn” from *formal* structures to *natural* structures. The sound patterns of this music are in fact natural structures, meaning that they form a connection with our more general experience of the world.

These natural structures are *aesthetic –*in the sense of aesthesis– as they belong to our broader experience of the world through our senses. This music offers patterns that are not *about* the world but portray the human experience of the world: by listening to these pieces, we are not *observing* the world but our modes of *acting* in the world. It is not that we are listening again to the world, it is that –through this music– we access the very structures of individual (of the listener and the artist) and communal experience acquired through surviving, growing and enacting in the world. This music provides access to the structures at the core of our wider experiential life and so has an additional cognitive potential compared to both other music and real-world sound material. This cognitive potential has a particular meaning to us precisely because the fundamental structures involved in how we experience the world are being activated.

While the aesthetic dimension of human meaning-making, in Dewey’s sense of the term (1934/1987), is at the heart of an integral experience where stimuli are richly cross-modal, sound-based music distills the essential elements of this experience. It represents a unique case study because it allows us to observe the fundamental nature of shared patterns across different modalities. The study of how this music is perceived provides instances of *cross-modal* patterns.

**Figure 5:**
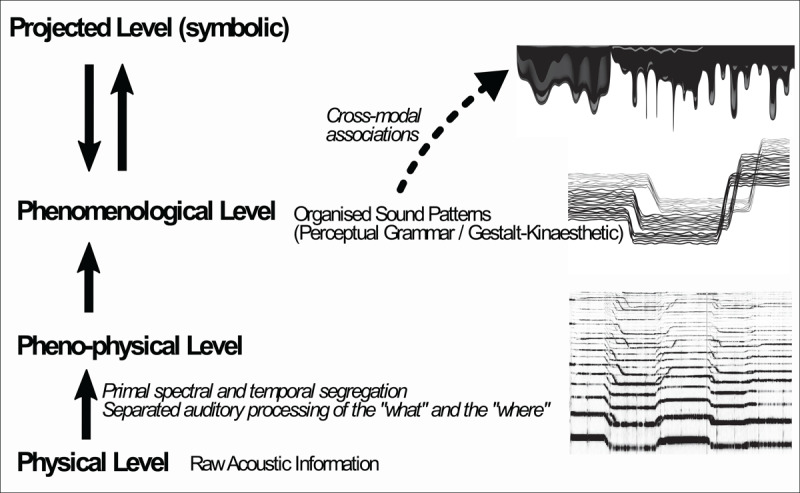
Schematic representation of the multi-level pathway which connects the physical level to the symbolic level (Petitot, 2011).

The world of sounds, *i.e*. this virtual domain emerging from the spectrotemporal qualities of acoustic stimuli, is a phenomenological level generated from the pheno-physical properties of acoustic stimuli (Petitot, 2011, pp. 50–51). Petitot explains the passage from the external micro-physical level –in our case, a level of raw acoustic information– to the pheno-physical level where macro-structures emerge (Figure 5):

When a system possesses many levels of organization, the higher “macro” level is causally reducible to the lower ones, yet at the same time its structures can be largely independent from the underlying “micro” dynamics. It benefits from a certain objective autonomy (ibid. 2011, p. 50).

The macro-structures of the pheno-physical level of auditory perception (and in particular of music listening) come from primitive segregation, both temporal and spectral, which allows the listener to group some acoustic elements and discard others in such a way that the chaotic aural environment becomes simpler and useful to us (Bregman, 1990). Moreover, we independently process spatial and non-spatial properties of acoustic stimuli (Alain et al., 2001) and these neurophysiological streams account separately for the identification of sonic content (pitch, spectral and temporal issues) and its external spatial location (based on interaural time and phase differences). Therefore, at a pheno-physical level we find macro-structures, such as the intrinsic sonic content, divided into separated streams, levels or layers, and the acoustic information accounting for external information, crucial in the case of everyday listening.

This pheno-physical level precedes and informs the phenomenological level, which, in the case of sound-based music, is the domain inhabited by sound patterns which no longer carry spatiotemporal information concerning the external physical world but rather their own spatiotemporal dimensions (Wanke & Santarcangelo, 2021; Imberty, 2005; Bergson, 1922/1998). At a phenomenological level, the sound patterns are now “arranged” (front/back, up/down…) in such a way that also depends on the higher symbolic level (projected level). At the projected level, everyone makes different associations based on their cultural values and practices, music-historical conventions, and prior experiences. Within the participants of the survey, for instance, the essential pattern of sample #3 evoked a cross-modal association with the visual and tactile experience of a viscous substance. Therefore, in this morphodynamic model, the phenomenological level of sound-based music is the locus of cross-modal patterns and is able to prompt cross-modal associations.

## Conclusions

This paper shows how powerful the application of morphodynamic theory to sound-based music can be, and it is the starting point from which to explore the potential of sound-based music to be understood within a connectionist model. The sound patterns of this music are found at a phenomenological level, which fits perfectly with a connectionist model: this level has physical origins (emergent), it is formal but not symbolic, it is topologically and geometrically formal but not logically formal.

If this proposal about the potential of sound to trigger meaning in itself may recall somehow a formalist paradigm (Hanslick), the very sense of this idea is connectionist: music is a human production, and sound-based music shows us better than other genres that meaning goes far beyond conceptual and propositional content to include our bodily experience. Sound-based music plays a key role as the *mean term* in a connectionist model, it is the interface between organized sounds and our sensorimotor apparatus (body), our brain, our cultural and social conventions, and our prior experiences. The structures of this music are then like emerging Gestalts, which, governed by essential syntactic relations, impact us at an implicit level. They are not formal assemblages of symbolic elements, but rather natural aesthetically organised Gestalts.

## Data Accessibility Statement

The author has no data accessibility statement to declare.

## Footnotes

While certain studies (Huron, 2006, p. 351) do not even deal with experimental and unconventional music practices, others talk about a “sabotage” of common perceptual and cognitive schemes (Snyder, 2001) or, by not responding to the proposed theories, raise doubts about the validity of such music (Lerdahl, 2001; Lerdahl & Jackendoff, 1983, p. 297).

In “+/–” (1996), for instance, Ryoji Ikeda makes use of electronic sounds outside of the audible frequency range.

For a recent analysis of the piece, please refer to (Hasegawa, 2015; Massoud, 2017).

A tritone is an interval which can create a bewildering effect in perception, being perceived at times as ascending or descending (Deutsch, 1986).

Here, I refer to the episode of *in vain* beginning at bar 240 where natural harmonic series of B, A, G, F-sharp, E-flat, D-flat, C and again B are presented sequentially (Haas, 2000).

Post-spectral pieces commonly include a compositional technique that goes beyond sound spectrum analysis and reunites timbral and harmonic elements with other elements of music.

The overall preference of trained listeners for dynamic linear drawings is significant (i.e. Pearson’s Chi-squared test with Yates’ continuity correction gives c^2^ = 5.534, p = .019). Binary logistic regression of related variables, i.e. the type of drawing and listener training, gives a non-significant model-Chi^2^ (Model- c^2^(1) = 5.793, p = .0161), while training appears a good predictor variable (b = 0.56, z = 2.456, p = .0141). Frequency Pearson’s *r* correlation between training and types of drawing is however very weak (*r* = .1014, t(593) = 2.48, p = .01328).
